# A single-centre, retrospective study of the incidence of invasive fungal infections during 85 years of autopsy service in Brazil

**DOI:** 10.1038/s41598-021-83587-1

**Published:** 2021-02-17

**Authors:** Kátia Cristina Dantas, Thais Mauad, Carmen D. Saldiva de André, Ana Luiza Bierrenbach, Paulo Hilário Nascimento Saldiva

**Affiliations:** 1 Department of Pathology, University of São Paulo-School of Medicine (FMUSP), São Paulo, Brazil; 2 University of São Paulo-Institute of Mathematics and Statistics, São Paulo, Brazil; 3 Research and Education Institute (IEP), Hospital Sírio-Libanês, São Paulo, Brazil; 4 Institute of Advanced Studies, University of São Paulo, São Paulo, Brazil

**Keywords:** Microbiology, Diseases, Medical research

## Abstract

Autopsy continues to play an essential role in monitoring opportunistic fungal infections. However, few studies have analysed the historical trends of fungal infections in autopsies. Here, we analyse available data on fungal infections obtained from autopsy reports during 85 years of autopsies performed by the largest autopsy service in Brazil. All invasive fungal infections presented in autopsy reports between 1930 and 2015 were included. Of the 158,404 autopsy reports analysed, 1096 involved invasive fungal infections. In general, paracoccidioidomycosis (24%) was the most frequent infection, followed by candidiasis (18%), pneumocystosis (11.7%), cryptococcosis (11%), aspergillosis (11%) and histoplasmosis (3.8%). Paracoccidioidomycosis decreased after the 1950s, whereas opportunistic fungal infections increased steadily after the 1980s during the peak of the AIDS pandemic. The lung was the most frequently affected organ (73%). Disseminated infection was present in 64.5% of cases. In 26% of the 513 cases for which clinical charts were available for review, the diagnosis of opportunistic fungal infections was performed only at autopsy. Our unique 85-year history of autopsies showed a transition from endemic to opportunistic fungal infections in São Paulo, Brazil, reflecting increased urbanization, the appearance of novel diseases, such as AIDS in the 1980s, and advances in medical care over time.

## Introduction

Autopsies have mirrored the epidemiological and clinical-laboratory evolution of the diagnosis of fungal infections, which are caused by endemic and opportunistic fungi. Since 1940, the diagnosis of fungal infections has dramatically increased with the widespread use of antibiotics, immunosuppressants, and invasive medical technologies. Currently, fungal pathogens remain a major cause of morbidity and mortality in immunodeficient patients^1–5^.

In Brazil, only limited information on mortality due to fungal infections can be obtained from the Brazilian Unified Health System database^6^. However, this data source probably underestimates the number of fungal infections that contributed to death because of disparities in health care access in this country. In 2011, Giocomazzi et al.^7^ estimated that more than 3.8 million individuals in Brazil, specifically patients with malignant cancers, transplantation, asthma, previous tuberculosis, or HIV infection and those living in areas endemic for highly pathogenic fungi, may have invasive fungal infections. Fungal infections usually affect socially vulnerable populations, with large public health consequences^7^. A lack of proper medical care is associated with increased mortality; for example, in low-/medium-income countries, cryptococcosis has high mortality rates, ranging from 43 to 65%, though rates in high-income countries remain at 10%^8^.

São Paulo is the largest city in Brazil. In the last century, São Paulo transformed from a semirural city of 500,000 inhabitants to a megacity of 12 million habitants. In the 1950s, with increased industrialization, a large internal migration of people from disadvantaged parts of the country contributed to increases in the population and resultant non-planned urbanization. Paracoccidioidomycosis was the most prevalent endemic mycosis in this area, with decreasing trends over the decades. A total of 1853 deaths due to paracoccidioidomycosis were recorded in Brazil between 1986 and 2006, representing 51% of the total number of deaths due to fungal infections^9–12^. In the last three decades, there has been an important decrease in mortality from paracoccidioidomycosis in the state of Sao Paulo, probably due to increased urbanization^13,14^.

Several autopsy studies have demonstrated an increase in the rate of fungal infections in recent decades. These studies were performed by groups from Asia (China, Japan, India, and Thailand), Europe (Germany and Italy), and Argentina, as well as Brazil. Most studies were established in centres with low autopsy rates and were conducted during a limited time span^5,15–18^. Furthermore, these studies showed important disagreement between *pre*- and post-mortem diagnoses^5,15,16,18–21^.

The Hospital das Clinicas of São Paulo University Medical School is the largest tertiary hospital in the country.

The purpose of this study was to analyse uniquely available data pertaining to invasive fungal infections obtained from 158,404 autopsy reports of the Department of Pathology of Sao Paulo University Medical School during the last 85 years. We hypothesized that a decrease in endemic fungal infections and an increase in opportunistic fungal infections occurred over the decades, paralleling city urbanization and medical advances. In addition, in the last 15 years, we evaluated pre- and post-mortem diagnoses of the detected fungal infections at autopsy.

## Results

Overall, 158,404 autopsies were performed between 1930 and 2015. The total number of hospital autopsies, sex and age stratified by decade are presented in Table 1. Autopsy rates decreased over the decades. During 1946–1995, the autopsy rates varied between 51 and 100%. After 1997, institutional changes in autopsy policies and lack of interest of the medical staff in academic autopsies decreased steadily, with an autopsy rate of 10% in 2015^22^.

**Table 1:** Numbers of hospital autopsy rates, sex and age by period (decade): total and fungal infection autopsies.

Autopsies			Sex N (%)				Age N (%)					
Periods	No. of autopsies (N)	Autopsy range (%)	Male	%	Female	%	Newborns (1–29 days)	1 month-14 years	15–29 years	30–44 years	45–59 years	60 years +
1930–1935	4569	nd	2961	64.8	1608	35.19	933 (20)	1305 (29)	687 (15)	669 (15)	787 (17)	188 (4)
1936–1945	14,718	nd	9019	61.27	5699	38.72	1532 (10.4)	4450 (30.2)	2813 (19)	1826 (12.4)	3086 (21)	1011 (7)
1946–1955	17,922	472–1511 (60–99)	11,365	63.41	6557	36.58	7026 (39)	1479 (8)	2355 (13)	1919 (11)	4131 (23)	1012 (6)
1956–1965	32,943	1438–3770 (62–98)	18,501	56.16	14,442	43.83	5035 (15)	10,753 (32)	3836 (12)	4513 (14)	6769 (20)	2037 (6)
1966–1975	40,500	1443–2254 (67–86)	23,538	58.11	16,962	41.88	7921 (19)	4325 (11)	5375 (13)	12,450 (31)	6481 (16)	3948 (10)
1976–1985	14,073	1288–1774 (83–100)	7741	55	6332	45	1952 (14)	2449 (17)	1601 (11.4)	2009 (14)	4862 (34.5)	1200 (9)
1986–1995	14,518	1714–3186 (51–89)	8636	59.48	5882	40.51	1052 (7)	1127 (7.8)	1445 (10)	2123 (14.6)	5879 (40.5)	2892 (20)
1996–2005	12,713	2366–2921 (30–64)	7007	55.11	5706	44.8	1763 (14)	1471 (11.6)	1652 (13)	2429 (19)	2922 (23)	2476 (19.4)
2006–2015	6448	2788–5137 (10–30)	3465	53.73	2983	46.26	985 (15)	687 (11)	570 (9)	716 (11)	2033 (31)	1457 (23)
Total	158,404		92,233	58.22	66,171	41.77						

Autopsies showing invasive fungal infection			Sex N (%)				Age N (%)					
Periods	No. of opportunistic fungal infections (N)	Incidence (%)	Male	%	Female	%	Newborns (1–29 days)	1 month–14 years	15–29 years	30–44 years	45–59 years	60 years +
1930–1935	5	0.10	5	100	0	0	0 (0)	0 (0)	1 (20)	3 (60)	1 (20)	0 (0)
1936–1945	32	1.86	28	87.5	4	12.5	0 (0)	2 (6.2)	12 (37.5)	9 (28.1)	9 (28.1)	0 (0)
1946–1955	60	0.33	51	85	9	15	0 (0)	3 (5)	26 (43.3)	25 (41.7)	5 (8.3)	1 (1.7)
1956–1965	103	0.31	78	75.7	25	24.3	12 (11.6)	5 (4.80	21 (20.4)	35 (34)	19 (18.4)	11 (10.7)
1966–1975	86	0.21	67	78	19	22	6 (7)	5 (5.8)	22(25.6)	27 (31.4)	17 (19.7)	9 (10.4)
1976–1985	30	0.21	27	90	3	10	1 (3.3)	0 (0)	7 (23.3)	7 (23.3)	11 (36.7)	4 (13.3)
1986–1995	67	0.46	55	82	12	18	3 (4.4)	1 (1.4)	14 (20.8)	24 (36)	17 (25.4)	8 (12)
1996–2005	384	3.0	255	66.4	129	33.6	31 (8)	20 (5.2)	62 (16.1)	128 (33.3)	79 (20.6)	64 (16.7)
2006–2015	329	5.1	190	58	139	42	24 (7.3)	17 (5.1)	36 (11)	58 (17.6)	99 (30)	95 (29)
Total	1096		756	68.97	340	31.02						

*Nd* no data, *d* days, *m* months, *y* years, *N* number, *%* percentage

During this period, 1,096 fungal infections (0.7% of all autopsies) were identified. Of these cases, 206 (19%) were classified as undefined, without identification of the fungal agent.

From 1932 to 1999, there were 145,181 autopsies and 377 (0.2%) fungal infections. From 2000 to 2015, there were 13,223 autopsies and 513 (4%) fungal infections. Of the 513 fungal infections identified after 2000, we were able to retrieve 378 (74%) clinical charts with available information.

**Figure 1:**
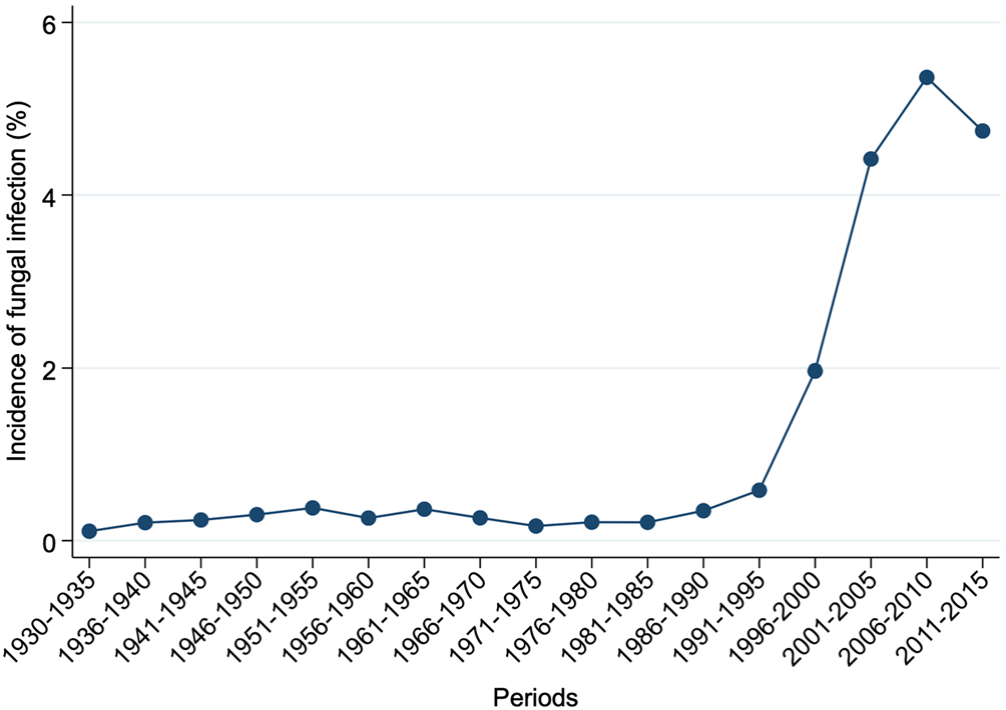
Annual evolution of the incidence of fungal infections among all death cases subjected to autopsy.

The proportion of fungal infections at autopsy varied from 0.1 to 5.1% across the 85-year period, as depicted in Fig. 1. After a long plateau of proportions below 0.5%, a sharp increase was observed from 1986 to 1995, and steady increases were observed over the subsequent 5-year periods, with a slight decrease in the last period.

Comparative analysis was performed only between the year of the first description of fungal infections in an autopsy report of this study and the first description in the literature, in Brazil and worldwide. Histoplasmosis and chromomycosis were first described in autopsy reports in 1946, candidiasis and mucormycosis were first described in autopsy reports in 1956, cryptococcosis was first described in 1954, pneumocystosis was first described in 1990, and aspergillosis was first described in 1996 (Table 2).

**Table 2:** Comparison of the year of the first description of fungal infections in the database and the first description in the literature, in Brazil and worldwide, when data were available.

		First described in the literature	First described in Brazilian studies	First described in autopsy studies	First described in this study
Paracoccidioidomycosis		–	1908^26,27^	1946^28^	1932
Candidiasis		1771^36^	–	1771^36^	1956
Pneumocystosis		1909^45^	1940^45^	1940^45^	1990
Cryptococcosis		1894^49^	1940^49^	–	1954
Histoplasmosis		1902^52^	1939^52^	1905^52^	1946
Aspergillosis		1842^44^	–	1842^44^	1996
Chromomycosis		1911^54^	–	1911^54^	1946
Mucormycosis		1876^56,57^	–	–	1956

Data were obtained from PubMed, the website https://www.cdc.gov/fungal/diseases, and Google Scholar in the English, Spanish and Portuguese languages.

**Figure 2:**
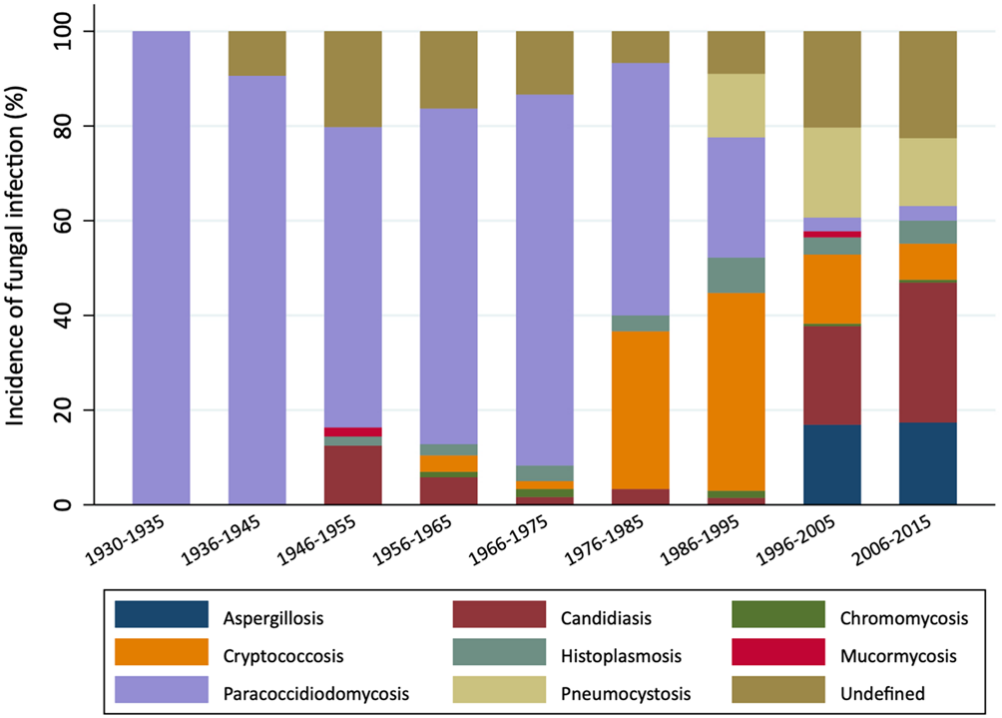
Proportional incidence of fungal infections by genus in autopsy reports between 1930 and 2015.

The analysis of the 1096 cases of fungal infection revealed that the most frequent infections in autopsies throughout the entire period were paracoccidioidomycosis (262, 24%) and candidiasis (198, 18%), followed by pneumocystosis (129, 11.7%), cryptococcosis (123, 11.2%), aspergillosis (122, 11.2%), histoplasmosis (42, 3.8%), chromomycosis (7, 0.6%), and mucormycosis (7, 0.6%). Fungal infections without identified pathogens accounted for 19% of all cases. The proportional distribution across the study period is shown in Fig. 2 and Table 3.

**Table 3:** Characteristics of fungal infections in autopsy reports by genus.

A. Characteristics of fungal infections in autopsy reports between 1930 and 2015 by genus											
Fungal infections		Aspergil (n = 122; 11.2%)	Cand (n = 198; 18%)	Chromo (n = 7; 0.6%)	Crypto (n = 123; 11.2%)	Histo (n = 42; 3.8%)	Mucor (n = 7; 0.6%)	Paraco (n = 262; 24%)	Pneumo (n = 129; 11.7%)	Undef (n = 206; 19%)	Total (n = 1096; 100%)
Sex	Female	55 (45)	88 (44)	0 (0)	30 (24.4)	15 (36)	5 (71)	31 (12)	40 (31)	76 (37)	340 (31)
	Male	67 (55)	110 (56)	7 (100)	93 (75.6)	27 (64)	2 (29)	231 (88)	89 (69)	130 (63)	756 (69)
Age group	Newborns	1 (1)	43 (22)	0 (0)	0 (0)	4 (9.5)	0 (0)	0 (0)	2 (2)	23 (11.2)	73 (6.7)
	1 month–14 years	15 (12)	20 (10)	0 (0)	1 (1)	2 (4.7)	1 (14)	10 (4)	2 (2)	6 (2.9)	57 (5.2)
	15–29 years	18 (15)	16 (8)	2 (29)	26 (21)	7 (16.7)	3 (43)	71 (27)	23 (18)	32 (15.5)	198 (18)
	30–44 years	33 (27)	32 (16)	1 (14)	48 (39)	7 (16.7)	0 (0)	82 (31)	60 (46)	48 (23.3)	311 (28.4)
	45–59 years	35 (29)	33 (17)	2 (28)	30 (24)	15 (35.7)	2 (29)	70 (27)	34 (26)	44 (21.4)	265 (24.2)
	60 years +	20 (16)	54 (27)	2 (29)	18 (15)	7 (16.7)	1 (14)	29 (11)	8 (6)	53 (25.7)	192 (17.5)

B. Characteristics of pre-mortem and post-mortem diagnoses of fungal infections in autopsy reports between 2000 and 2015 by genus											
Diagnoses (n; %)	Aspergil (n = 115; 22.4%)	Cand (n = 176; 34.3%)	Chromo (n = 4; 0.8%)	Crypto (n = 64; 12.5%)	Histo (n = 29; 5.6%)	Mucor (n = 5; 1%)	Paraco (n = 17; 3.3%)	Pneumo (n = 103; 20%)	Total (n = 513; 100%)		
Pre-mortem^a^	82 (71.3)	130 (74)	3 (75)	49 (76.5)	23 (79.3)	4 (80)	15 (88)	72 (70)	378 (74)		
Post-mortem^b^	33 (28.7)	46 (26)	1 (25)	15 (23.4)	6 (20.7)	1 (20)	2 (12)	31 (30)	135 (26)		

	Underlying disorders in patients diagnosed pre-mortem with fungal infections	Aspergil (n = 82; 21.7%)	Cand (n = 130; 3.5%)	Chromo (n = 3; 0.8%)	Crypto (n = 49; 13%)	Histo (n = 23; 6.1%)	Mucor (n = 4; 1%)	Paraco (n = 15; 4%)	Pneumo (n = 72; 19.1%)	Total (n = 378; 100%)	
Clinical charts	AIDS	7 (8)	20 (15.4)	0 (0)	20 (41)	10 (44)	1 (25)	1 (6.6)	51 (71)	110 (29)	
	Cancer	44 (54.6)	28 (21.5)	0 (0)	2 (4)	3 (13)	1 (25)	1 (6.6)	4 (5)	83 (22)	
	Diabetes	2 (2.4)	28 (21.5)	0 (0)	1 (2)	5 (22)	2 (50)	0 (0)	6 (8)	44 (12)	
	Preterm infants	0 (0)	22 (17)	0 (0)	0 (0)	0 (0)	0 (0)	0 (0)	2 (3)	24 (6)	
	Respiratory diseases	2 (2.4)	18 (14)	0 (0)	10 (20.4)	3 (13)	0 (0)	12 (80)	7 (10)	52 (14)	
	Transplant	12 (14.6)	6 (4.6)	1 (33.3)	4 (8.1)	2 (7)	0 (0)	1 (6.6)	2 (3)	28 (7.3)	
	Other disease	15 (18)	8 (6)	2 (66.6)	12 (24.5)	0 (0)	0 (0)	0 (0)	0 (0)	37 (9.7)	
Organs	Lung	76 (92.6)	67 (51.5)	3 (100)	31 (63.2)	21 (91.3)	4 (100)	15 (100)	61 (84.7)	278 (73.5)	
	CNS	7 (8.6)	37 (28.4)	3 (100)	46 (94)	22 (95.6)	3 (75)	11 (73.3)	15 (20.8)	144 (38)	
	Spleen	6 (7.4)	36 (27.7)	2 (66.6)	5 (10.2)	2 (8.7)	0 (0)	1 (6.6)	13 (18)	65 (17.2)	
	Kidneys	8 (9.5)	38 (29.2)	0 (0)	12 (24.5)	1 (4.3)	0 (0)	10 (66.6)	18 (25)	87 (23)	
	Liver	30 (3.7)	66 (50.7)	0 (0)	17 (34.7)	5 (21.7)	1 (25)	10 (66.6)	21 (29.1)	150 (39.7)	
	Other	3 (3.7)	19 (14.6)	0 (0)	5 (10.2)	5 (21.7)	0 (0)	3 (20)	1 (1.4)	36 (9.5)	
Disseminated^a^		62 (75)	81 (62)	2 (66)	30 (61)	14 (61)	2 (50)	9 (60)	44 (61)	244 (64.5)	

Newborns 1–29 days; *m* month, *y* year, *N* number, *%* percentage, *a* autopsies confirmed the diagnosis made pre-mortem, *b* diagnosis confirmed by autopsy only, *AIDS* acquired immunodeficiency syndrome, *Aspergil* aspergillosis, *Cand* candidiasis, *Chromo* chromomycosis, *Crypto* cryptococcosis, *Histo* histoplasmosis, *Mucor* mucormycosis, *Paraco* paracoccidioidomycosis, *Pneumo* pneumocystosis, *Undef* undefined fungal infections.

^a^Percentage of cases with disseminated disease.

### Total autopsy cases × sex and total fungal infections × sex

Fifty-nine percent of the autopsied population in the study period were men. Over time, the percentage of autopsies performed on females increased. Male predominance was also observed in autopsies associated with fungal infections (69% of the cases). Male predominance was observed across all infections except for mucormycosis; however, the number of patients with this particular infection was low (Tables 1 and 3).

### Fungal disease × age group

**Figure 3:**
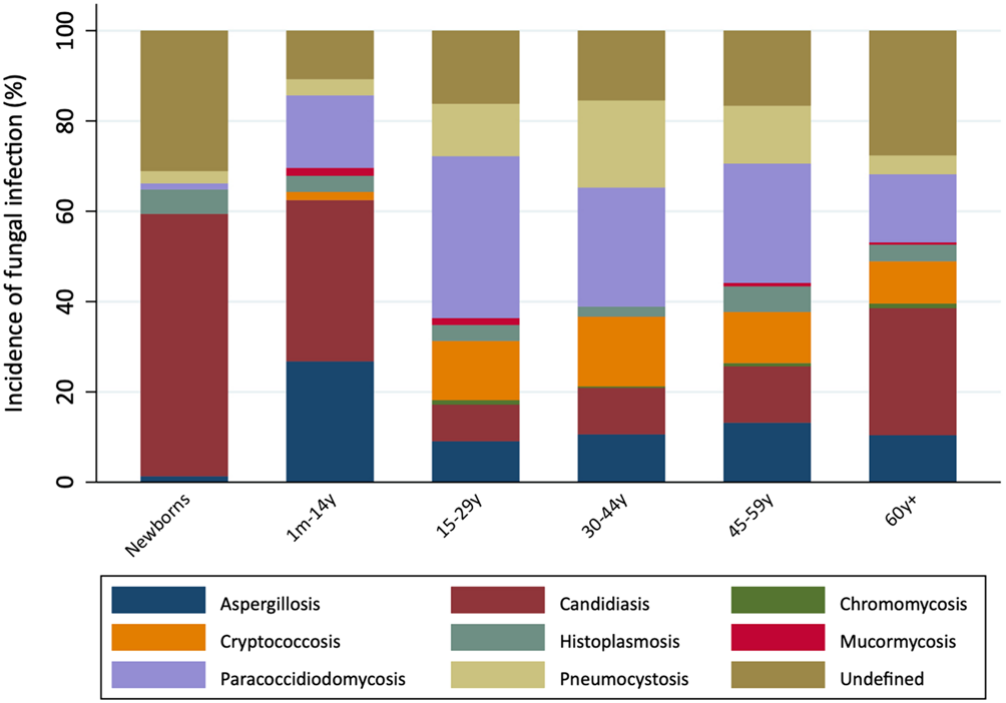
Age distribution of fungal infections in autopsy reports between 1930 and 2015.

The pathogen distribution varied by age group (Table 3 and Fig. 3). The proportion of candidiasis was higher among newborns and elderly adults than among those in the other age groups. The proportions of those with undefined aetiology were also higher among newborns and elderly adults than among those in the other age groups. Paracoccidioidomycosis and cryptococcosis were predominant among adolescents and young adults. Pneumocystosis was most commonly observed in adults aged 30–44 years, while aspergillosis was most common among adults aged 30–44 and 45–59 years. Over the years, there was an increase in the median age of patients (Table 1).

### Fungal disease × comorbid condition (n = 378 cases with clinical charts available, 2000–2015)

Most of the observed fungal infections were opportunistic and affected individuals with comorbid conditions prone to immunosuppression.

Those with no known prior comorbidities tended to have a history of previous hospitalization or antibiotic use or were elderly individuals (Table 3). AIDS was the most frequently observed comorbidity, affecting 29% of all individuals and up to 71%, 41%, and 44% of those with pneumocystosis, cryptococcosis and histoplasmosis, respectively. Cancer was the second most frequently occurring comorbidity, affecting 22% of all individuals and up to 54.6% and 21.5% of those with aspergillosis and candidiasis, respectively. Of the few individuals with mucormycosis, 50% had diabetes. The most common infection among premature newborns was candidiasis (17%). Various fungal infections occurred among transplant recipients, but the most frequently observed was aspergillosis.

Of the 15 paracoccidioidomycosis cases with available clinical information, eight (23.5%) had concomitant tuberculosis, one had AIDS (6.6%), one had cancer (6.6%), and four (23.5%) were considered juvenile cases.

### Fungal infections × organ involvement (n = 378 cases with clinical charts available, 2000–2015)

Fungal pathogens were detected in more than one organ by anatomopathological examination. Considering all 378 cases, the lungs were the most frequently affected organ (73.5%), followed by the liver (39.7%), central nervous system (CNS) (38%), kidneys (23%), and spleen (17.2%). Other organs affected by fungal pathogens accounted for 9.5% of all cases (leg, bone, uterus, prostate, ganglia, lymph nodes, skin, adrenal glands, thyroid, peritoneum, colon, stomach, oesophagus, tongue, pancreas, bladder, pharynx, and bone marrow). Many cases involved more than one affected organ, which is why the sum of the percentages in the column in Table 3 does not add up to 100%. Two organs were affected in 60% of the cases, and aspergillosis was the infection with the highest frequency (Table 3).

Table 3 also shows a different profile in terms of the organ affected for each pathogen. The lungs were the most commonly affected organ for all the diseases except for candidiasis and histoplasmosis. For cryptococcosis, the CNS was the most affected organ, followed by the lungs.

### Concomitant infections

Most of the concomitant fungal infections were identified in patients with AIDS (9, 8%), respiratory diseases (3, 6%) and cancer (3, 3.6%). The following associations were found: candidiasis and cryptococcosis (3, 13%), candidiasis and aspergillosis (4, 17.4%), and candidiasis and pneumocystosis (3, 13%). Additionally, associations between candidiasis and histoplasmosis, cryptococcosis and pneumocystosis, cryptococcosis and histoplasmosis, aspergillosis and paracoccidioidomycosis, aspergillosis and histoplasmosis, aspergillosis and mucormycosis, pneumocystosis and histoplasmosis, cryptococcosis and aspergillosis, and pneumocystosis and candidiasis were less frequently reported than the previously mentioned associations (1, 4.35%).

### Pre-mortem versus post-mortem diagnoses for each fungal pathogen

Fungal infections were identified during pre-mortem evaluations in 74% of the cases and confirmed with post-mortem analyses, while in 135 cases (26%), they were detected only in post-mortem analyses (pathological finding only) (Table 3).

## Discussion

In this study, we describe the history of invasive fungal infections diagnosed at autopsy from 1930 to 2015 in a large university hospital in São Paulo, Brazil. The data from 1,096 autopsies with invasive fungal infections over 85 years showed a predominance of paracoccidioidomycosis that peaked in 1966–1975 and subsequently decreased. However, in the early 1980s, the proportion of fungal infections associated with immunosuppressive conditions rose steadily, especially in patients with AIDS, respiratory diseases, cancer, transplant and diabetes. To our knowledge, this is the first autopsy study analysing fungal infections over such a long-time span and large number of cases.

Our data showed that there was an increase in the mean age of patients over time, possibly reflecting the increase in life expectancy in Brazil (30 years since 1940)^23^. Increased age was also associated with an increased incidence of fungal infection. Males had higher rates of fungal infection than females, similar to the findings of Lacaz et al.^24^. Paracoccidioidomycosis infection had a major predominance in males in our study. In addition to the lack of occupational exposure, hormonal factors have been discussed as protective factors in women^25^. A higher proportion of mucormycosis was detected among women; however, the number of cases was too low to draw any conclusions.

The two earliest cases of paracoccidioidomycosis were reported in 1908 by Adolpho Lutz in Brazil^26^, who described the clinical manifestations and anatomopathological findings of the disease. In 1928, Almeida and Lacaz introduced the name Paracoccidioides, and Almeida named the fungus *Paracoccidioides brasiliensis* in 1930^27^. In our study, the first autopsy report was described in 1932; however, in the literature, the first autopsy record was not until 1946^28^.

The proportion of paracoccidioidomycosis-related deaths, which was higher in the early study period than in the late study period, has considerably decreased in Brazil in the last few decades^10^_._ Several factors may have contributed to this decrease, as follows: (1) improvements in the diagnostic capacity of paracoccidioidomycosis; (2) the use of new effective drugs (azoles); (3) the widespread use of fungicides in crops that indirectly reduced the inhalation of environmental fungi; and (4) increased urbanization^29–33^. Paracoccidioidomycosis may also be associated with immunosuppression and other concomitant infections. In our sample, the most frequently associated condition was tuberculosis (3%), followed by chronic obstructive pulmonary disease (COPD). Indeed, tuberculosis may affect up to 15% to 20% of patients with paracoccidioidomycosis^34^.

The presence of juvenile paracoccidioidomycosis (acute/subacute form) was observed in 23.5% of deaths; this value was higher than that described in the 2018 Brazilian guidelines for the clinical management of paracoccidioidomycosis^35^. *Paracoccidioides* sp. infection has been observed in a wide age range of patients, from two-year-old children to elderly patients, although most patients are adults^30,31^.

Candidiasis was first described in 1771 by Rosen von Rosenstein^36^; in the present study, the first autopsy report was published in 1956. Invasive candidiasis was the second most frequent pathogen identified among our cases and the disease that most frequently occurred in recent years (2006–2015)^7,15^. Candidaemia is the most commonly occurring form of invasive candidiasis and is associated with unacceptably high mortality^37^. In our study, the increasing incidence of invasive candidiasis over the decades was associated with underlying diseases such as cancer, diabetes, and AIDS^15,38^. In premature newborns and in the 1 month-14-year age group, the highest proportion of deaths was attributed to candidiasis^5^. In newborns, candidiasis occurs partly due to the immaturity of the immune system and to the virulence mechanisms of the fungus^39^. Similarly, candidiasis was the most prevalent infection in elderly individuals^40^. Several factors, such as a high frequency of comorbidities and age-related physiological changes, may explain the high mortality in elderly individuals.

The first diagnosis of invasive aspergillosis in this study was performed in 1996, and the incidence of aspergillosis (11.2%) was lower than that in the literature, even in the most recent years^14,15,41,42^. Such a low incidence may be explained by difficulties in identifying the pathogen by conventional histological methods and the low incidence of the disease until the 1990s^43^. In 1959, aspergillosis was frequently described in birds and domestic animals and was not considered common in humans^44^. Deaths related to aspergillosis were observed for the first time in the 1990s in association with cancer and stem cell transplantation^6^. It is believed that the incidence of invasive aspergillosis is increasing, and many cases are diagnosed only at autopsy^43^. In our study, the most frequently associated condition in patients with aspergillosis was cancer (54.6%).

Pneumocystosis was first described in 1909 by Carlos Chagas, but it was recognized as a human pathogen in the 1940s after it was observed in the lungs of malnourished preterm infants^45^. Since 1960, pneumocystosis has been recognized as a cause of pneumonia in adults with immunosuppression^46^. Since 1980, there has been a dramatic increase in *Pneumocystis jirovecii* infections associated with AIDS. In our study, *P. jirovecii* infection first appeared in the 1990s and was associated with AIDS-related deaths in 71% of the cases^47^. In our series, only two cases of were observed in preterm infants in our series*P. jirovecii* infection in preterm infants were observed.

The first case of human cryptococcosis was described in 1894 by Otto Busse and Abraham Buschke, who described the infection in a 31-year-old woman with tibial injury^48^. Before the appearance of AIDS, cryptococcosis was considered a rare disease related to deficiencies in cellular immunity^24^. The first cryptococcosis-related deaths in São Paulo were reported in the 1940s. With the AIDS pandemic and the use of immunosuppressive drugs, there has been an increase in cryptococcosis incidence since the 1980s, and the disease currently ranks as the fourth most frequent cause of opportunistic infection in HIV-positive patients^49^. In our study, cryptococcosis was first reported in an autopsy in 1954 and showed an incidence of fungal infection of 14%, with increasing frequencies after the 1980s and a high frequency of CNS involvement^38^. In the AIDS cases in our study, pneumocystosis and cryptococcosis infections were the most commonly found fungal infections, similar to observations by other authors^47,50,51^.

*Histoplasma* was discovered in 1905 by Samuel T. Darling, but it was described as causing widespread infection only in the 1930s. Before that, many cases of histoplasmosis were mistakenly attributed to tuberculosis^52^. *Histoplasma* spp. accounted for an incidence of fungal infection of 5% in our cases. The first cases appeared in the 1940s, but their proportion increased only after the 1980s. Cases of meningitis caused by histoplasmosis appeared in immunocompromised individuals, especially those with AIDS, and patients receiving corticosteroids or other forms of immunosuppressive therapy or cytotoxic chemotherapy. In our study, cases of disseminated histoplasmosis were described in 44% of AIDS patients^53^.

We identified very few cases of chromomycosis and mucormycosis. Chromomycosis is a subtropical/tropical cutaneous disease that was first described in 1911 in São Paulo. Disease dissemination is rare. We identified seven cases over the span of 85 years, with the first case occurring in 1946. Of the three cases in which full autopsy records were available, we identified brain involvement in all three, supporting the theory that this infection is neurotrophic^54,55^. Pulmonary mucormycosis was first described in 1876 by Furbringer^56^, and in a classic review in 1955, Baker^57^ thoroughly described all mucormycosis cases previously reported. In our study, mucormycosis was first described in an autopsy report in 1956. Mucormycosis invasive infection (7 cases) was observed in patients with diabetes (50%) and those receiving chemotherapy for lymphoproliferative diseases or other neoplasms. Both infection prevalence rates in autopsy increased in the 1990s, presenting a different profile compared with that reported by Suzuki et al. (2013)^20^, in which chromomycosis and mucormycosis showed consistent prevalence rates over the years.

Sixty-four percent of the studied cases presented disseminated forms, and the most affected organs were the lungs; the exception was histoplasmosis, which had a higher frequency of CNS involvement than lung involvement^20,21^. The analysis of autopsy reports from 2000 showed that a pre-mortem diagnosis of fungal infection occurred in 74% of the cases, and paracoccidioidomycosis was the most frequently identified infection^7,16,19^. The most common fungal infections identified in post-mortem diagnoses were pneumocystosis, aspergillosis and candidiasis^7,15,16^.

Comparative analysis of pre-mortem and post-mortem diagnostic findings showed that fungal infections were diagnosed only at autopsy in 26% of the cases, which emphasizes the importance of autopsy for clinical monitoring, even in current practice^58–63^. These numbers may be even higher because undefined fungal infections identified at autopsy were not included in this percentage.

Our study had some limitations. First, the cases from 1930 to 1999 were analysed with summarized information from autopsy reports containing only information on the underlying disease and the leading cause of death, and some cases were probably missed. Another limitation was that a change in the profile of the autopsied populations occurred after 1975, which may have influenced the number of endemic fungal infections observed in the period. Unfortunately, due to technical limitations and the large amount of data, we were unable to retrieve information on the number of key underlying diseases to serve as denominators. Moreover, we did not review histological slides to determine which histological criteria were used for each case during the different periods, as autopsy techniques and pathologists’ knowledge about fungal infections improve over time, which impacted our data. This phenomenon might explain the higher incidence of invasive fungal infections after 2000 in our study. Our main objective was to provide a historical perspective of fungal infections seen in our service in the city of São Paulo over a period of 85 years. Despite these limitations, the large number of cases over a period of 85 years revealed interesting historical trends in fungal infections.

Nineteen percent of the cases were suspected to have a clinical diagnosis of fungal infection, but the fungal pathogens were no longer classified in the post-mortem diagnosis. Our data also show an increase in undefined cases in the two most recent decades. A potential reason could be the progressive use of new immunosuppressive therapies for transplantation or cancer that can hinder fungal detection. Furthermore, the low rates of concomitant infections in this sample could be attributed to the previous use of antifungal therapy, especially in patients with AIDS. Our data suggest that post-mortem fungal cultures should be more widely performed in autopsies, which are not routine in this department. In addition, the use of molecular techniques in this study could have improved the identification of pathogens and their distribution in organs and could even have aided in the identification of new species in this study.

In summary, analysis of this autopsy series contributes to our understanding of the distribution of different fungal infections over time as well as the historical aspects, epidemiology and diagnosis of fungal infections. In addition, the findings provide information on an extremely relevant issue in clinical practice: the rate of pre-mortem diagnosis, which can be considered a quality control tool. With the dramatic decrease in conventional autopsies worldwide, minimally invasive autopsy (MIA) should be considered a feasible alternative, as previous studies in adults and children have shown good accuracy in identifying fungal infections using this procedure^64–67^.

## Methods

### Ethical statement

This study was approved by the research ethics committee of the Clinical Hospital of the University of São Paulo of Medicine (HCFMUSP) (CAPPesq n#3.930.342; CAAE: 28882220.0.0000.0068). The CAPPesq waived the need for informed consent for this study because it was not possible to reach participants or family members during the period studied. The study declaration was performed in accordance with the relevant guidelines and regulations of the ethics committee of HCFMUSP following the approval CAPPesq # 3.930.342. This retrospective study was carried out using information from autopsy reports and clinical charts from patients who died in the academic hospital.

### Autopsy reports

This retrospective study was conducted at the Department of Pathology at the São Paulo University Medical School using autopsy reports and clinical charts from patients who died in the municipality of São Paulo (including the academic hospital) from 1930 to 1974 and from patients who died exclusively in the academic hospital from 1975 onward. It was not possible to differentiate the two populations during the period 1930–1974 via the autopsy reports. Up to 1997, there was an institutional policy of very high rates of autopsy requests, which changed afterward.

From 1930 to 1999, digitized reports contained only information about the basic disease and main cause of death (http://www.acervopatologia.fm.usp.br). Since 2000, full autopsy reports have been available for digital consultation. Autopsies were performed by residents under the supervision of senior pathologists since 1968, when the residency programme began in this service. A thorough macroscopic and microscopic examination was performed using histochemically stained specimens and immunohistochemistry when necessary. All autopsy reports with a diagnosis of deep fungal infection were retrieved based on the pathological finding of fungi in at least one deep tissue. Post-mortem cultures were not routinely performed. We selected autopsy reports with a tissue diagnosis of candidiasis, aspergillosis, pneumocystosis (described in the anatomopathological analysis as *Pneumocystis carinii, P. jirovecii)*, mucormycosis, chromomycosis, paracoccidioidomycosis, blastomycosis, *P. brasiliensis*, South American blastomycosis, cryptococcosis and histoplasmosis. Cases with the presence of non-classifiable fungi in the autopsy reports were considered undefined. Fungal infections were considered disseminated when two or more organs were affected.

### Revision of clinical charts

Clinical charts of patients who died after 2000 and for whom autopsy identified a deep fungal infection were retrieved. From the available charts, we extracted demographic data, underlying diseases/comorbidities, and the presence/absence of a pre-mortem diagnosis of deep fungal infection based on positive serology, direct mycology, positive cultures or tissue demonstration. If the pathogen was identified during only the autopsy procedure (tissue infection with fungi), the case was considered to have a post-mortem diagnosis; otherwise, it was characterized as a pre-mortem diagnosis.

### Data presentation

The incidence of fungal infections was computed as the total number of cases at each 5-year interval divided by the total number of autopsies in the same period. Age was also categorized into six groups: newborns (1–29 d), 1 month-14 years, 15–29 years, 30–44 years, 45–59 years, and 60 years or older. The characteristics of fungal infections in autopsy reports between 1930 and 2015 were analysed every 10 years.

### Statistical analysis

Data were stored in MS Excel 2010, and descriptive statistical analyses were performed using Stata 15 (Stata Corp. LP, College Station, TX, USA).

Numbers and proportions or medians and interquartile intervals were used to categorize fungal diseases by sex, age group, time period, comorbid condition, affected organs, and pre- and post-mortem diagnoses.

The overall incidence of fungal infection was calculated as the number of deaths due to fungal disease in proportion to all deaths recorded in the database during the same period. The incidence of infection for each fungal pathogen was calculated as the number of deaths caused by a specific fungal disease as a proportion of all deaths caused by fungal diseases during the same period.
